# Turning Data Optimization of Titanium Alloy Produced by Casting and DMLS

**DOI:** 10.3390/ma18245583

**Published:** 2025-12-12

**Authors:** Ksenia Latosińska, Wojciech Zębala

**Affiliations:** Department of Production Engineering, Faculty of Mechanical, Cracow University of Technology, 31-155 Cracow, Poland; wojciech.zebala@pk.edu.pl

**Keywords:** titanium alloy, DMLS, turning, specific cutting force, optimization

## Abstract

In manufacturing processes, both material processing methods and the resulting microstructure play a fundamental role in determining material behavior during component fabrication and subsequent service conditions. Materials produced by additive manufacturing exhibit a unique microstructure due to the rapid heating and solidification cycles inherent to the process, distinguishing them from conventionally cast counterparts and leading to differences in mechanical and functional properties. This article presents problems related to the longitudinal turning of Ti6Al4V titanium alloy elements produced by the casting and powder laser sintering (DMLS) methods. The authors made an attempt to establish a procedure for determining the optimal parameters of finishing cutting while minimizing the specific cutting force, taking into account the criterion of machined surface quality. In the course of the experiments, the influence of the cutting data on the cutting force values, surface roughness parameters, and chip shape was examined. The material hardening state during machining and the variability of the specific cutting force as a function of the cross-sectional shape of the cutting layer were also tested. The authors presented a practical application of the proposed optimization algorithm. It was found that by changing the shape of the cross-section of the cutting layer, it was possible to carry out the turning process with significantly reduced specific cutting force (from 2300 N/mm^2^ to 1950 N/mm^2^) without deteriorating the surface roughness.

## 1. Introduction

Titanium occurs in nature in the form of various compounds and minerals, constituting about 0.43–0.61% of the Earth’s crust mass [1]. Thanks to its availability and specific physical and chemical properties, it has become very popular in modern industry. The main disadvantages of titanium are the high costs of obtaining and subsequently processing it [2,3]. Titanium and its alloys belong to the S material group. Titanium is characterized by high relative strength, low sensitivity to temperature changes, and good resistance to chemical factors and, subsequently, is highly resistant to corrosion. Its specific properties include high biocompatibility (titanium is non-toxic and hypoallergenic), low thermal expansion, high melting point, and that it is non-magnetic [3,4,5]. Due to its properties, titanium is widely used in the aerospace industry. It is estimated that about 80% of the production of this material is dedicated to the needs of the aviation industry. It is used for gas turbine elements, chassis and suspension structural elements, and all kinds of joints [6,7]. Titanium alloys are commonly used in the chemical, fuel, and energy industries in such elements as heat exchangers, pipelines, and chemical reactors [2]. In the automotive industry, they are mainly used for mechanically and thermally loaded engine and turbocharger components, exhaust systems, and suspension components [8].

Titanium alloys are very difficult to machine due to their very high strength and plasticity. These properties cause titanium to generate large amounts of heat in the cutting process. The low thermal conductivity of titanium alloys causes heat to accumulate in the cutting zone and transfer to the cutting edge [9,10,11]. On the other hand, the increase in temperature in the machining zone may reduce cutting resistance, which is beneficial from a quality and energy perspective [12]. When machining titanium alloys, the chip is generally formed in segments, which causes the tool to be loaded with cyclic mechanical and thermal stress, and the frequency and amplitude of these stresses directly depend on the cutting parameters. This leads to a faster wear of the tool and the initiation of cracks and chipping [13].

Sintered carbides, high-speed steels, diamond, and ceramics are the most often used materials in tools for machining titanium. The greatest difficulty is that they react chemically with the titanium which leads to their accelerated wear. The use of a CBN (cubic boron nitride) produces the best results in terms of surface quality, tool life, and cutting forces [9].

Machining is one of the most frequently used and highest energy-consuming production processes; therefore, it is necessary to research and develop optimization methods that reduce the energy consumption of this process [14]. In the case of machining, especially the turning process, specific methods are used to reduce the cutting forces while increasing productivity and optimizing the entire process. The authors used various tools during turning to determine their effects on the machining of titanium alloys. Da Silva et al. [15] showed that the use of coatings on cutting tools lowered the coefficient of friction. Lindvall et al. [16] analyzed the wear mechanism of the tools made of materials with higher strength (in particular PCD and pCBN) during the machining of titanium alloys.

Many authors have investigated the optimization of machining processes in terms of machining time, volume of material removal, and machining strategies [17,18,19]. An improvement in the machinability of titanium alloys can be achieved by applying various dry machining methods [20], as well as by using cutting fluids such as minimum quantity lubrication (MQL) [21,22] or high-pressure cooling (HPC) [23]. Zhng and Liu [24] assessed the surface topography of the Ti6Al4V alloy after dry turning. They showed an increase in the parameters Ra and Rz and that the roughness profile of the machined surface changed with an increase in feed. Sartori et al. [25] investigated the surface integrity of Ti6Al4V alloy after cryogenic and dry treatment. The surface of the processed material deteriorated in cryogenic conditions, but at the same time, fewer surface defects were observed.

When machining titanium alloys, the main area of research is the influence of parameters such as cutting speed, feed, and depth of cut on the quality of the machined surface, determination of cutting forces, and chip formation and breakage [26].

Many research papers have addressed the optimization of titanium alloys machining with various methods and mathematical models. The authors developed a turning process optimization model that took into account the following factors influencing the machining: cutting force (*Fc*) tool life (*T*), surface roughness (*Ra*), material removal rate (*Qv*), and the process of chip formation and breakage [27,28,29].

The main methods of forming titanium alloys are forging, casting, and sintering, but thanks to the sintering technology, both energy and raw materials can be saved. In addition, sintered parts are characterized by good mechanical properties and precision and are easy to shape into different forms [30]. In recent years, one of the most popular methods of producing powder-based materials is the additive manufacturing (AM) method. It allows for the production of very complex and precise elements with high accuracy and a high degree of structure densification [31,32,33].

The aim of the article is to develop a method for optimizing the machining process of Ti6Al4V titanium alloy made by two different methods (casting, DMLS printing) while maintaining the best quality of the machined surface and process efficiency.

## 2. Materials and Methods

The investigations were carried out on cylindrical specimens manufactured by casting and laser sintering (DMLS) from the titanium alloy Ti-6Al-4V, commercially known as Grade 5. The DMLS material was produced using a Renishaw Am 250 system. The following process parameters were applied during specimen fabrication: laser power of 400 W, scan speed of 289 mm/s, exposure time of 75 µs, point distance of 75 µm, hatch spacing of 0.165 mm, and layer thickness of 60 µm. The mechanical properties and chemical composition of both materials are presented in Table 1 and Table 2.

During the experiments, measurements of the components of the cutting forces, surface roughness of the machined surface, temperature in the cutting zone, and analysis of the chips formed during the process were conducted. Surface roughness measurements were performed using a Talysurf Intra 50 profilometer (Taylor Hobson, Leicester, UK). For the acquisition and analysis of cutting force components, a measurement system was employed based on a Kistler 9257B dynamometer and a 5070B amplifier (Winterthur, Switzerland). Temperature acquisition was performed using an infrared thermal camera (FLIR SC 620, Wilsonville, OR, USA). The maximum permissible temperature (T_limit) was defined based on [34,35] and corresponds to the threshold above which tool wear or material degradation is accelerated.

The turning tests were carried out using CNMG120408-MF4 TS2500 cutting inserts supplied by SECO (Fagersta, Sweden), mounted in a PCLNR 2020K-12 toolholder manufactured by ISCAR (Tefen, Israel). The cutting parameters were selected within the range recommended by the tool manufacturer, i.e., *ap* = 0.5–1.0 mm and *f* = 0.08–0.25 mm/rev. A constant cutting speed of *vc* = 30 m/min was applied. The experimental design and the range of cutting parameters are presented in Table 3. All experiments were performed in triplicate for each set of test conditions. The reported results correspond to the arithmetic mean of the three repetitions and its standard deviation.

## 3. Results and Discussion

In accordance with the adopted experimental plan, measurements of the components of the total cutting force during longitudinal turning of cast and additively manufactured Ti-6Al-4V titanium alloy were performed. The influence of the cross-sectional area of the machined layer *A_D_* on the values of the total cutting force components—main *Fc*, feed *Ff*, and passive *Fp*—as well as on the specific cutting resistance, was analyzed. The cross-sectional area of the machined layer was considered as a function of the variable cutting parameters, namely feed *f* and depth of cut *ap*.

The specific cutting resistance *kc* is defined as the ratio of the main cutting force *Fc* to the cross-sectional area of the machined layer *A_D_* (1).

(1) $$k_{c}=\frac{F_{c}}{A_{D}}=\frac{F_{c}}{f\;*\;a_{p}}[N/{mm}^{2}]$$

where: *Fc*—main cutting force (N),

*A_D_*—cross-sectional area of the machined layer (mm^2^),

*k_c_*—specific cutting resistance (MPa) (N/mm^2^).

**Figure 1:**
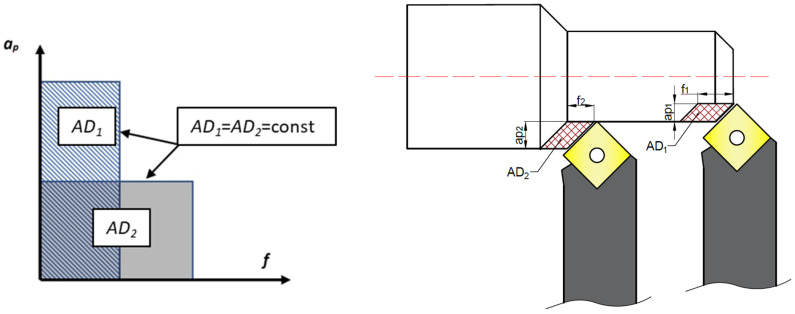
A simplified diagram of the cross-section shapes of the cutting layer while maintaining a constant cross-section value for turning with *Κ_R_* = 90°.

For materials from different groups (with varying chemical compositions and microstructures), the specific cutting force may differ even at the same tensile strength. Those differences are the result of the different microstructures of the cast and DMLS samples. Casting leads to slower cooling rates, resulting in larger grains, coarse dendritic structures, and potential segregation. DMLS involves extremely rapid melting and solidification, producing fine dendritic or cellular structures, characteristic crystallo-graphic textures, and local microstructural gradients [36]. The value of the specific cutting force depends on the cross-sectional area *A_D_*. To determine the influence of the cross-sectional shape of the removed layer on the specific cutting forces *kc*, experimental studies were conducted. Cutting-force tests were carried out for various cross-sectional shapes of the removed layer while maintaining a constant cross-sectional area, *A_D_* = const. Figure 1 schematically illustrates the variable shape of the cut-layer cross-section at constant area. The change in cross-sectional shape results from adjusting the proportions of the dimensions that define the removed layer—namely, the feed *f* and the depth of cut *ap*.

**Figure 2:**
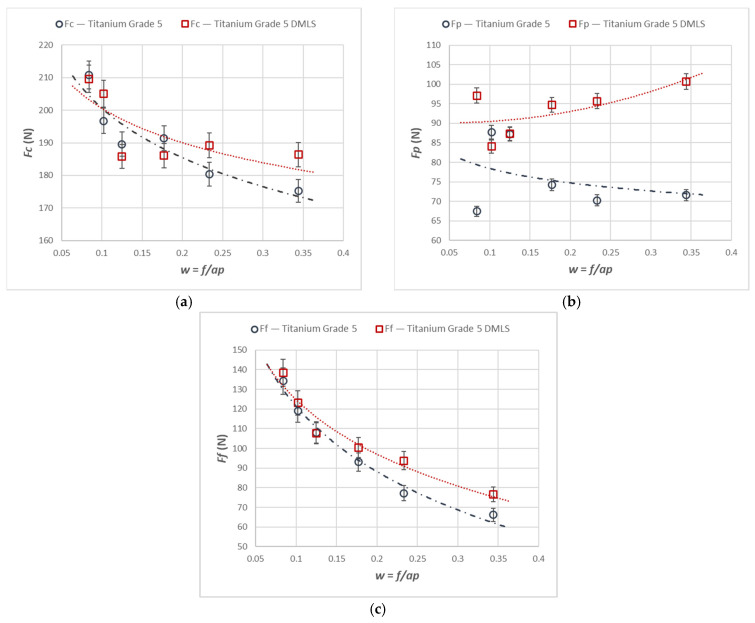
The characteristics of the components of the total cutting force for turning with an *A_D_* = 0.08 mm^2^ constant cross-section of the cutting layer for the printed and cast Ti6Al4V alloy. (**a**) *Fc* component, (**b**) *Fp* component, (**c**) *Ff* component.

Figure 2 shows the variation in the total cutting forces *Fc*, *Ff*, and *Fp* as a function of the constant cross-sectional area of the removed layer, *A_D_* = 0.08 mm^2^, during turning of the cast and laser-printed Ti6Al4V alloy.

Based on an analysis of the results, it can be stated that all the components of the total cutting force during turning of the additively manufactured material were higher than those obtained for the cast material. These differences, however, were not very large. It was observed that the differences increased above *w* > 0.20, which describes the ratio of feed *f* to depth of cut *ap*. The trends in the main cutting force *Fc* were very similar for both the cast and the additively manufactured alloy, whereas differences appeared in the *Fp* and *Ff* components. The greatest discrepancies in the force components occurred at the smallest cut depths and the largest feed values.

**Figure 3:**
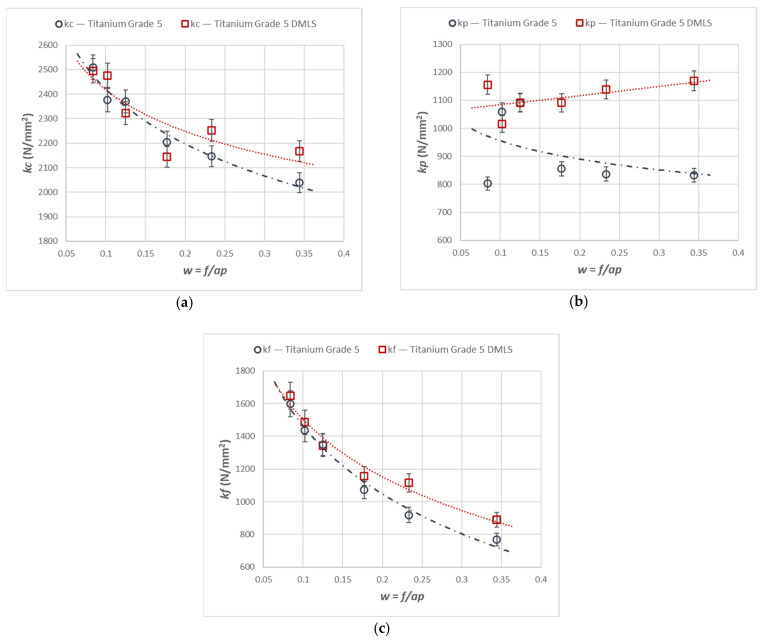
Cutting resistance relationships for the components *Fc*, *Ff*, and *Fp* for turning with an *A_D_* = 0.08 mm^2^ constant cross-section of the cutting layer for the printed and cast Ti6Al4V alloy, characteristics of (**a**) *kc*, (**b**) *kf*, and (**c**) *kp*.

Similar trends to those observed for the force components were found for the corresponding specific cutting forces. Figure 3 presents the variations in the specific cutting forces *kc*, *kf*, and *kp* corresponding to the components *Fc*, *Ff*, and *Fp* for the turning of the printed and cast Ti6Al4V titanium alloy.

**Figure 4:**
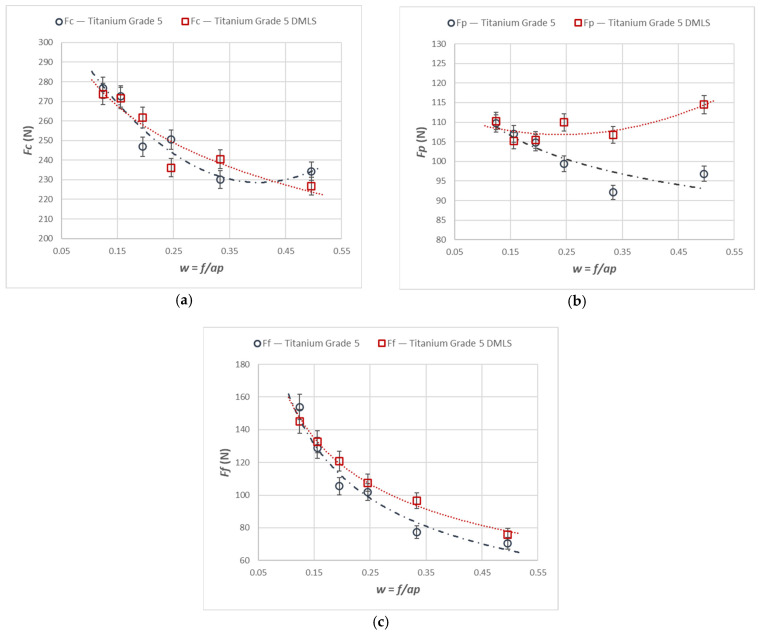
The characteristics of the components of the total cutting force for turning with an *A_D_* = 0.12 mm^2^ constant cross-section of the cutting layer for the printed and cast Ti6Al4V alloy, characteristics of (**a**) *Fc* component, (**b**) *Fp* component, and (**c**) *Ff* component.

Figure 4 shows the variation in the force components *Fc*, *Ff* and *Fp* as a function of the constant cross-sectional area of the removed layer, *A_D_* = 0.12 mm^2^, during turning of cast and laser-printed Ti6Al4V.

A comparative analysis of the cutting-force values for increasing cross-sectional area *A_D_* revealed that all components rose overall while maintaining a nearly constant difference between the cast and printed material. A decrease in the *Fc* and *Ff* components was observed, with increasing values of the parameter w for both cross-section values. In contrast, the *Fp* component increased for every cross-section considered.

**Figure 5:**
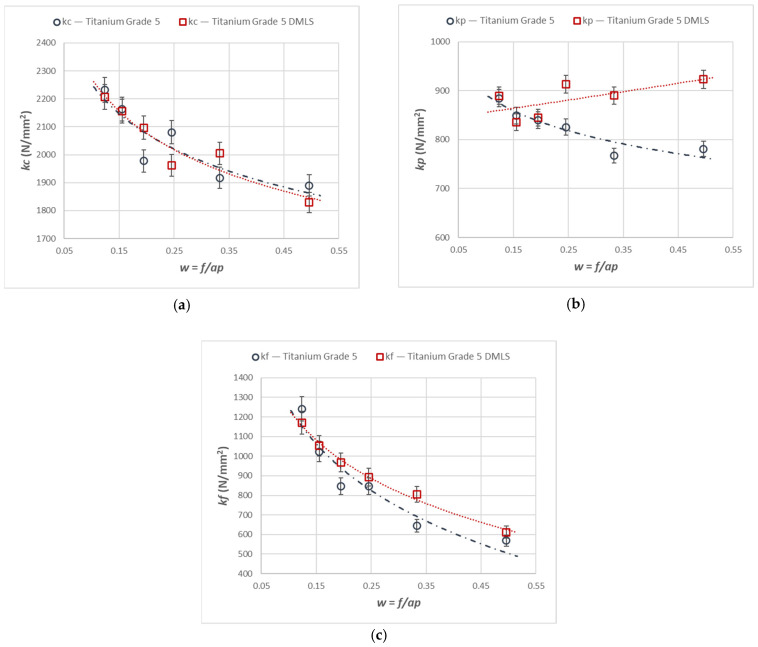
Cutting resistance relationship for the components *Fc*, *Ff*, and *Fp* for turning with a constant *A_D_* = 0.12 mm^2^ cross-section of the cutting layer for the printed and cast Ti6Al4V alloy, characteristics of (**a**) *kc*, (**b**) *kf*, and (**c**) *kp*.

Figure 5 presents the variations in the specific cutting forces *kc*, *kf*, and *kp* corresponding to the force components *Fc*, *Ff*, and *Fp* for *A_D_* = 0.12 mm^2^ during turning of the printed and cast Ti6Al4V titanium alloy.

The specific cutting forces *kc* and *kf* decreased with an increase in parameter w for both analyzed cross-section areas. It should be noted that the values of the specific cutting forces for the larger cross-section (*A_D_* = 0.12 mm^2^) were lower than those for the smaller cross-section. This indicates that by modifying the shape of the cross-section of the removed layer, the turning process can be conducted at reduced specific cutting forces. This is relevant for selecting machining allowances and for conducting both finishing and roughing operations. However, it should be emphasized that changing the cross-section shape reduces the volumetric efficiency *Qv*, which increases the total cutting time *T_total_*.

**Figure 6:**
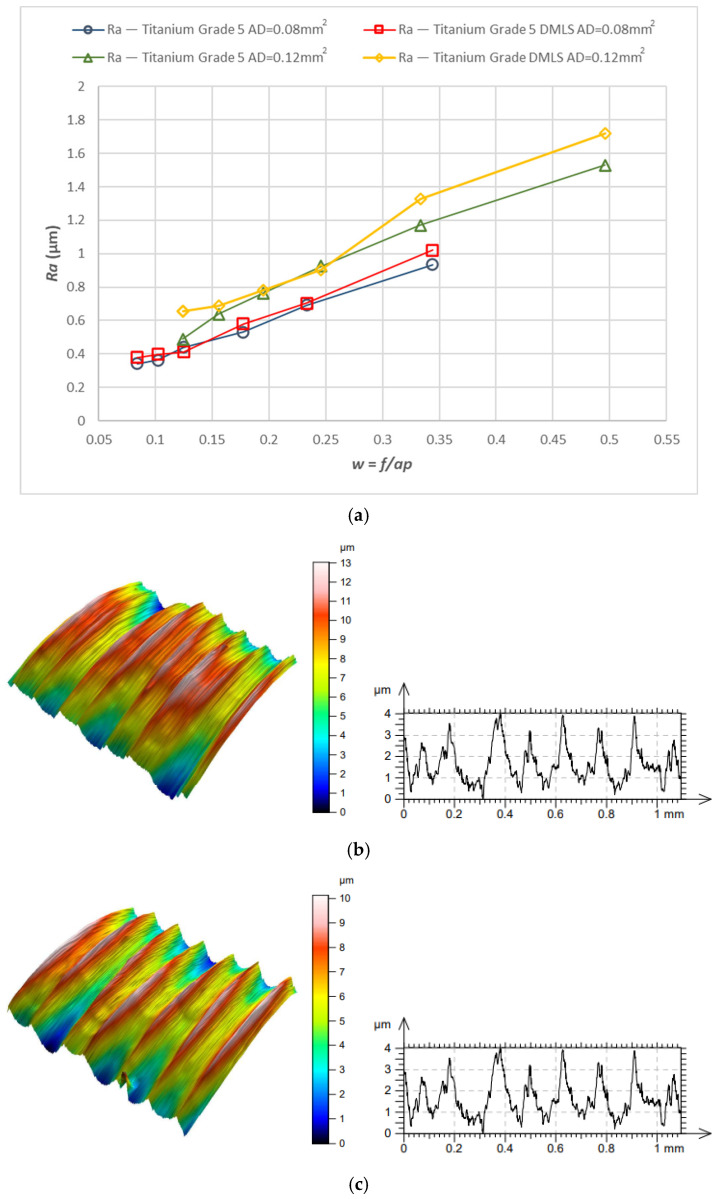
Surface roughness: (**a**) Ra roughness relationship for turning with a constant cross-section of the cutting layer (*A_D_* = 0.08 mm^2^ and *A_D_* = 0.12 mm^2^) for 3D-printed and cast Ti6Al4V alloy; (**b**) shape and profile—3D printed material, *w* = 0.24; (**c**) shape and profile—cast material, *w* = 0.24.

Figure 6 shows the surface roughness Ra obtained during turning of cast and 3D-printed Ti6Al4V. In all cases, surface roughness increased. This resulted from increasing the feed *f* in order to maintain a constant cross-sectional area, which necessarily reduced the depth of cut *ap*. It should be noted that for the cast material, Ra values were slightly lower than those for the laser-printed material at higher values of the parameter *w*.

Table 4 presents examples of chip morphologies obtained during the turning of the laser-sintered and cast alloys. In both cases—printed and cast—a long, ribbon-like spiral or snarled chips were observed. For both materials, the chip length was unacceptable, with *L_ch_* > 150 mm, because continuous long chips hinder chip evacuation, increase the risk of entanglement around the tool or workpiece, and may negatively affect surface quality and process safety [37].

## 4. Application of the Algorithm

**Figure 7:**
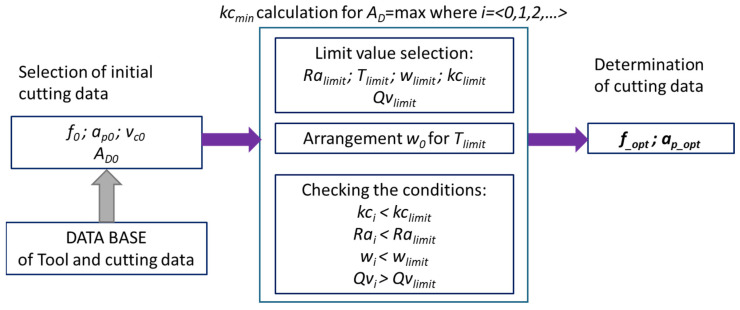
A simplified diagram of the procedure with the minimization of the actual cutting resistance.

The algorithm presented in Figure 7 illustrates the procedure for selecting cutting parameters in such a way as to achieve the minimum specific cutting force (*kc*) while maintaining the required technological, quality, and operational constraints. This process involves the iterative selection of cutting conditions and their verification against established limit values.

The procedure shown in Figure 7 proceeds through the following stages:

1. Selection of Limit Values

In the first stage, the limit values of key technological parameters are defined. These serve as the criteria for evaluating whether the process is performed correctly. This group includes:

- *Ra__limit_*—allowable surface roughness after machining;
- *T__limit_*—maximum permissible temperature in the cutting zone;
- *w__limit_*—limit value of the ratio of feed to depth of cut;
- *kc__limit_*—allowable value of the specific cutting force;
- *Qv__limit_*—minimum required volumetric efficiency of the process.

These limits constitute the reference point for subsequent analysis and are typically determined based on prior experimental studies, tool manufacturer recommendations, or technological requirements of the machining process. Each of those parameters are selected individually for the selected material and tool.

2. Determination of the Input Parameter Set

Based on the adopted temperature limit *T__limit_*, an initial set of input parameters *w_0_* are selected, corresponding to a specific set of cutting conditions. This set may include, among others, cutting speed *vc*, feed *f*, depth of cut *ap*, tool geometry, and properties of the machined material.

The aim of this stage is to define parameters that do not exceed the permissible temperature in the cutting zone while still enabling effective machining.

3. Verification of the Limiting Criteria

For the obtained parameter set *w_0_*, the actual values of the controlled quantities are calculated. The following conditions are then checked:

*kc*_*i*_ < *kc*__*limit*_

*Ra*__*i*_ < *Ra*__*limit*_

*w*__*i*_ < *w*__*limit*_

*Qv*__*i*_ > *Qv*__*limit*_

If all conditions are satisfied, the selected parameter set ensures a minimal specific cutting force while maintaining adequate surface quality, tool life, and process efficiency.

If at least one criterion is not met, the algorithm returns to the previous stage to modify the input parameters. This iterative process continues until a set fulfilling all constraints is identified.

The experiments demonstrated that selecting specific *f* and *ap* (ap)—more precisely, the ratio *w = f*/*ap*—affects the specific cutting force (*kc*), which in turn can reduce tool wear, lower energy consumption, and improve the precision of the final product. This approach also enables control over the surface roughness (*Ra*) within the desired range for finishing processes. These relationships are consistent across the materials tested in this study. Based on these findings, an algorithm was developed to predict the optimal combination of *f* and *ap*.

**Figure 8:**
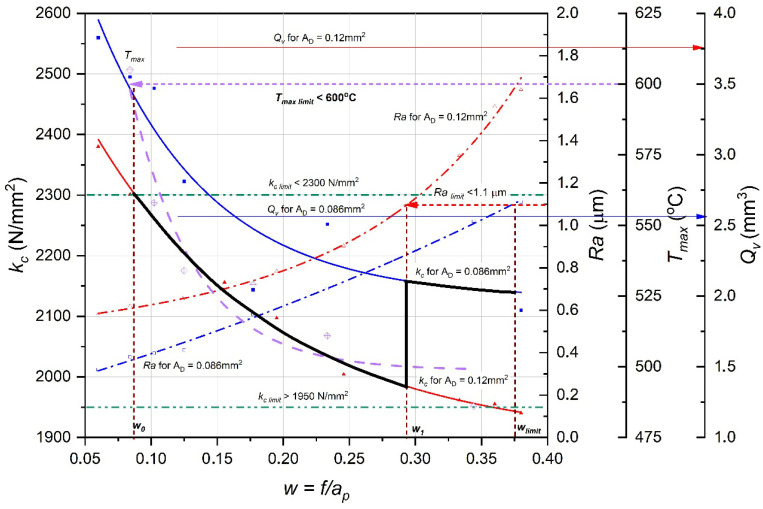
Conceptual diagram of the method for sample data.

Figure 8 illustrates the operation of the specific cutting force minimization algorithm (*kc*) using sample experimental data. The dependencies are shown as a function of the parameter *w* = *f*/*ap*, which represents the feed-to-depth-of-cut ratio. Changing this ratio directly influences the cutting force, temperature, surface roughness, and volumetric efficiency. Two cross-sectional areas of the removed layer were analyzed: *A_D_* = 0.086 mm^2^ and *A_D_* = 0.12 mm^2^.

The graph includes several dashed lines marking the limit values defined by the algorithm:

- *kc__limit_* = 2300 N/mm^2^ and 1950 N/mm^2^—allowable specific cutting force;
- *Ra__limit_* < 1.1 µm—allowable surface roughness;
- *T__max_limit_* < 600 °C—allowable cutting-zone temperature;
- *Qv__limit_*—minimum volumetric efficiency.

These values serve as criteria for evaluating the process in subsequent steps.

The point *w*_0_ (on the left side of the graph) represents the initial set of input parameters selected based on the temperature constraint *T__limit_*. In this region, the specific cutting force *kc* is very high (exceeding 2500 N/mm^2^), and the temperature approaches the upper allowable limit. This indicates that the cutting process is energetically inefficient, and thermal loads on the tool are excessive.

As the ratio *w* = *f*/*ap* increases, a decrease in the specific cutting force is observed. The value of *kc* decreases until reaching an optimum near point *w*_1_, where all limiting conditions are satisfied:

*kc*_*i*_ < *kc*__*limit*_

*Ra*__*i*_ < *Ra*__*limit*_

*T*__*max*_*i*_ < *T*__*limit*_

*Qv*__*i*_ > *Qv*__*limit*_

In this region (between *w*_0_ and *w__limit_*), the most favorable cutting parameters are achieved—minimal specific cutting force with the required surface quality and sufficient tool life.

The range between the vertical lines w_0_ and *w__limit_* represents the permissible cutting-parameter region in which all technological constraints are met. Point *w*_1_ can be considered the optimal operating point of the process, corresponding to the minimum specific cutting force while maintaining thermal safety and quality requirements.

From the presented diagram, it follows that as the ratio *w* = *f*/*ap* increases, the following occurs:

- The specific cutting force *kc* decreases;
- The maximum temperature *T__max_* also decreases;
- Surface roughness Ra increases slightly;
- Volumetric efficiency *Qv* increases.

Therefore, proper selection of the parameter w enables a compromise between energy consumption and machining quality. According to the proposed algorithm, optimal conditions are those that minimize kc while satisfying the constraints *Ra__limit_*, *T__limit_*, and *Qv__limit_*.

After reaching point w_1_, corresponding to the minimum specific cutting force for the given cross-sectional area *A_D_*, it is possible to transition to the next characteristic curve (indicated by the bold vertical line). This curve describes the relationship *kc(w)* for a smaller removed-layer cross-section.

By moving along this new curve in the direction of increasing w, without exceeding *w_limit*, the minimization procedure can again be carried out. Although this transition results in a reduction in volumetric efficiency *Qv* and a slight increase in *kc*, it also enables a more favorable feed-to-depth-of-cut ratio, yielding a more stable process, improved heat removal, and reduced load on the cutting edge.

Thus, the method supports multistage optimization: after reaching a local optimum for a given cross-section, one can proceed to a smaller *A_D_* and repeat the procedure to identify further improved cutting conditions.

The algorithm structure and logic are universal, but specific thresholds (e.g., force limits and recommended parameter ranges) are material-dependent and would need to be recalibrated for alloys with different mechanical or thermal properties.

## 5. Conclusions

Based on the obtained experimental results and the conducted analyses, the following conclusions can be drawn:

- The values of all components of the total cutting force during turning of additively manufactured material are similar to, or slightly higher than, those observed when turning cast material. A progressive increase in these differences was observed for parameters above *w* > 0.15. For *w* < 0.15, the cutting force values during turning of cast and laser-sintered material are comparable across all components.
- By modifying the cross-sectional shape of the machined layer, the turning process can be carried out at reduced cutting resistance. However, in the present case, in order to maintain the surface roughness requirement of Ra ≤ 1.1 µm, a slight increase in the specific cutting resistance *kc* is necessary.

The algorithm presented in this study is of an optimization-oriented nature and can be applied both in experimental analyses and in industrial practice. Its purpose is to identify a process operating point that ensures minimum specific cutting resistance without compromising surface quality or tool life. Through the use of an iterative procedure, it is possible to obtain an optimal compromise between machining efficiency, surface quality, and process economics.

The developed algorithm thus represents a practical tool supporting the design and optimization of machining parameters. It may be used during the planning phase of technological experiments as well as in decision-support systems for industrial machining processes. From the perspective of future research, the algorithm may be extended with an adaptive module utilizing real-time measurement data (e.g., cutting forces, vibrations, and temperature), thereby enabling dynamic parameter optimization and further improving process efficiency. 

## Figures and Tables

**Table 1:** Mechanical properties of the material Ti-6Al-4V for DMLS and cast parts.

Property	Value
Mechanical	
Tensile strength (MPa)	1000
Yield strength (Rp 0.2%) (MPa)	900
Hardness (HRC)	30
Elongation to break A (%)	15
Percent reduction in area after fracture (Z)	41

**Table 2:** Chemical composition of Ti-6Al-4V manufactured by casting and DMLS.

Element	Ti	Al	V	O	Fe	C	Mn	N	H
wt.(%)	rest.	5.5–6.75	3.5–4.5	0.2 max	0.3 max	0.06	0.05	0.05	0.01

**Table 3:** Test plan and range of parameters for cast and printed material.

No.	Coded Parameter			*A_D_* = 0.08 mm^2^		*A_D_* = 0.12 mm^2^	
	A	B	*vc* (m/min)	*f* (mm/rev)	*ap* (mm)	*f* (mm/rev)	*ap* (mm)
1.	1	1	30	0.084	1	0.124	1
2.	2	2	30	0.092	0.9	0.14	0.9
3.	3	3	30	0.1	0.8	0.156	0.8
4.	4	4	30	0.124	0.7	0.172	0.7
5.	5	5	30	0.14	0.6	0.2	0.6
6.	6	6	30	0.172	0.5	0.248	0.5

**Table 4:** Chips obtained during turning of laser-sintered and cast alloys.

Cross-Section of Cutting Layer	Titanium Grade 5	Titanium Grade 5 DMLS
*A_D_* = 0.08 mm^2^	*ap* = 1.0 mm, *f* = 0.084 mm/rev.	*ap* = 1.0 mm, *f* = 0.084 mm/rev.
	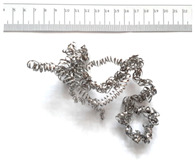	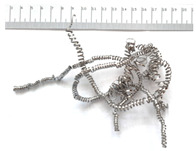
	*ap* = 0.8 mm, *f* = 0.100 mm/rev.	*ap* = 0.8 mm, *f* = 0.100 mm/rev.
	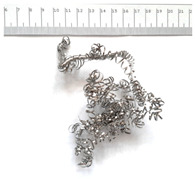	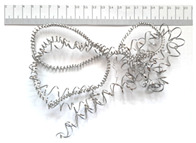
	*ap* = 0.5 mm, *f* = 0.172 mm/rev.	*ap* = 0.5 mm, *f* = 0.172 mm/rev.
	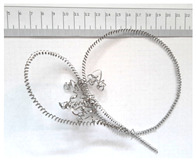	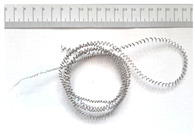
*A_D_* = 0.12 mm^2^	*ap* = 1.0 mm, *f* = 0.084 mm/rev.	*ap* = 1.0 mm, *f* = 0.084 mm/rev.
	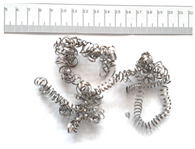	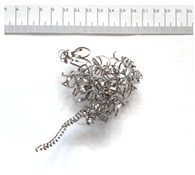
	*ap* = 0.8 mm, *f* = 0.100 mm/rev.	*ap* = 0.8 mm, *f* = 0.100 mm/rev.
	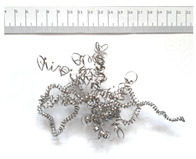	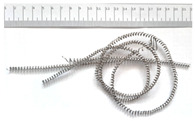
	*ap* = 0.5 mm, *f* = 0.172 mm/rev.	*ap* = 0.5 mm, *f* = 0.172 mm/rev.
	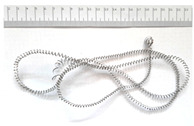	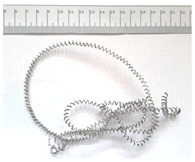

## Data Availability

The original contributions presented in this study are included in the article. Further inquiries can be directed to the corresponding author.

## References

[1] Chunxiang C., BaoMin B., Lichen Z., Shuangjin L. (2011). Titanium alloy production technology, market prospects and industry development. Mater. Des..

[2] Pushp P., Dasharath S.M., Arati C. (2022). Classification and Applications of Titanium and Its Alloys. Mater. Today Proc..

[3] Gupta K., Laubscher R.F. (2016). Sustainable machining of titanium alloys. A critical review. Proc. Inst. Mech. Eng. Part B J. Eng. Manuf..

[4] Schwartz A., Kossenko A., Zinigrad M., Danchuk V., Sobolev A. (2023). Cleaning Strategies of Synthesized Bioactive Coatings by PEO on Ti-6Al-4V Alloys of Organic Contaminations. Materials.

[5] Blakely-Milner B., Gradal P., Snedden G., Brooks M., Pitot J., Lopez E., Leary M., Berto F., du Plessis A. (2021). Metal additive manufacturing in aerospace: A review. Mater. Des..

[6] Shafir S., Rahime E., Sasahara H. (2012). Machinability of Titanium Alloys in Drilling, Titanium Alloys. Towards Achieving Enhanced Properties for Diversified Applications.

[7] Dudek Ł., Hryniewicz T., Rokosz K. (2016). Zastosowanie Tytanu i wybranych stopów tytanu w lotnictwie. Autobusy Tech. Eksploat. Syst. Transp..

[8] Sachdev A.K., Kulkarni K., Fang Z.Z., Yang R., Girshov V. (2012). Titanium for Automotive Applications: Challenges and Opportunities in Materials and Processing. J. Miner. Met. Mater. Soc..

[9] El-Hadad S., Elsayed A., Shi B., Attia H. (2023). Experimental Investigation on Machinability of Titanium Alloys with Different Microstructures. Materials.

[10] Siju A.S., Waigaonkar S.D. (2021). Effects of rake surface texture geometries on the performance of single-point cutting tools in hard turning of titanium alloy. J. Manuf. Process..

[11] Hosokawa A., Kosugi K., Ueda T. (2022). Turning characteristics of titanium alloy Ti-6Al-4V with high-pressure cutting fluid. CIRP Ann..

[12] Shetty P.K., Shetty R., Shetty D., Rehaman F., Jose T.K. (2014). Machinability Study on Dry Drilling of Titanium Alloy Ti-6-Al-4V using L9 Orthogonal Array. Procedia Mater. Sci..

[13] Seketi S., Odelros S., Östby J., Olsson M. (2019). Experimental Study of Wear Mechanisms of Cemented Carbide in the Turning of Ti6Al4V. Materials.

[14] Gutowski T., Dahmus J., Thirie A. Electrical energy requirements for manufacturing processes. Proceedings of the 13th CIRP International Conference on Life Cycle Engineering.

[15] Da Silva R.B., Machado Á.R., Ezugwu E.O., Bonney J., Sales W.F. (2013). Tool life and wear mechanisms in high speed machining of Ti-6Al-4V alloy with PCD tools under various coolant pressures. J. Mater. Process. Technol..

[16] Lindvall R., Bjerke A., Salmasi A., Lenrick F., M’Saoubi R., Stahl J.E., Bushyla V. (2023). Predicting wear mechanisms of ultra-hard tooling in machining Ti6Al4V by diffusion couples and simulation. J. Eur. Ceram. Soc..

[17] Rao R.V., Kalyanakar V.D. (2014). Optimization of modern machining processes using advanced optimization techniques. A review. Int. J. Adv. Manuf. Technol..

[18] Jawahir I.S., Wang X. (2007). Development of hybrid predictive models and optimization techniques for machining operations. J. Mater. Process. Technol..

[19] Löschner P., Niesłony P., Kołodziej S. (2025). Parameter Sensitivity Study of the Johnson–Cook Model in FEM Turning of Ti6Al4V Alloy. Materials.

[20] Sánchez Hernández Y., Trujillo Vilches F.J., Bermudo Gamboa C., Sevilla Hurtado L. (2018). Experimental parametric relationships for chip geometry in dry machining of the Ti6Al4V alloy. Materials.

[21] Pimenov D.Y., Mia M., Gupta M.K., Machado A.R., Tomaz I.V., Sarikaya M., Wojciechowski S., Mikołajczyk T., Kapłonek W. (2021). Improvement of machinability of Ti and its alloys using cooling-lubrication techniques: A review and future prospect. J. Mater. Res. Technol..

[22] Kumari S., Shah M., Modi Y., Bandhu D., Zadafiya K., Abhishek K., Saxena K.K., Msomi V., Mohammed K.A. (2022). Effect of Various Lubricating Strategies on Machining of Titanium Alloys: A State-of-the-Art Review. Coatings.

[23] Singh J., Gill S.S., Dogra M., Singh R. (2021). A review on cutting fluids used in machining processes. Eng. Res. Express.

[24] Zhang P., Liu Z. (2016). Modeling and prediction for 3D surface topography in finish turning with conventional and wiper inserts. Measurement.

[25] Sartori S., Bordin A., Ghiotti A., Bruschi S. (2016). Analysis of the surface integrity in cryogenic turning of Ti6Al4V produced by Direct Melting Laser Sintering. Procedia CIRP.

[26] Martínez E.G., Miguel V., Martínez A.M., Manjabacas M.C., Coello J. (2019). Sustainable Lubrication Methods for the Machining of Titanium Alloys: An Overview. Materials.

[27] Gupta M., Sood P.K., Singh G., Sharma V.S. (2017). Sustainable machining of aerospace material–Ti (grade-2) alloy: Modeling and optimization. J. Clean. Prod..

[28] Wang X., Da Z.J., Balaji A.K., Jawahir I.S. (2007). Performance-based predictive models and optimization methods for turning operations and applications: Part 3—Optimum cutting conditions and selection of cutting tools. J. Manuf. Process..

[29] Lisowicz J., Krupa K., Leksycki K., Rusinek R., Wojciechowski S. (2025). Analysis of Tool Wear in Finish Turning of Titanium Alloy Ti-6Al-4V Under Minimum Quantity Lubrication Conditions Observed with Recurrence Quantification Analysis. Materials.

[30] Wang Z., Tan Y., Ning L. (2023). Powder metallurgy of titanium alloys: A brief review. J. Alloys Compd..

[31] Guo M., Gu D., Xi L., Du L., Zhanga H., Zhanga J. (2019). Formation of scanning tracks during Selective Laser Melting (SLM) of pure tungsten powder: Morphology, geometric features and forming mechanisms. Int. J. Refract. Met. Hard Mater..

[32] Srivastava M., Jayakumar V., Udayan Y., Sathishkumar M., Muthu S.M., Gautam P., Nag A. (2024). Additive manufacturing of Titanium alloy for aerospace applications: Insights into the process, microstructure, and mechanical properties. Appl. Mater. Today.

[33] Schuch M., Hahn T., Herzig N., Bleckmann M. (2022). The Mechanical Behavior and Microstructure of Additively Manufactured Ti6AI4V for Different Material States and Loading Conditions. J. Dyn. Behav. Mater..

[34] Boyer R.R. (1996). An overview on the use of titanium in the aerospace industry. Mater. Sci. Eng..

[35] Champin B., Graff L., Armand M., Béranger G., Coddet C. (1980). Oxydation des alliages de titane au voisinage des températures d’utilisation dans les turbomoteurs. J. Less Common Met..

[36] Zębala W., Struzikiewicz G., Rumian K. (2021). Cutting Forces and Tool Wear Investigation during Turning of Sintered Nickel-Cobalt Alloy with CBN Tools. Materials.

[37] Dejian L., Chenbing N., Yougiang W., Zhu L. (2024). Review of serrated chip characteristics and formation mechanism from conventional to additively manufactured titanium alloys. J. Alloys Compd..

